# Multi-expert synthesis for versatile locomotion and manipulation skills

**DOI:** 10.3389/frobt.2022.970890

**Published:** 2022-09-28

**Authors:** Kai Yuan, Zhibin Li

**Affiliations:** ^1^ School of Informatics, The University of Edinburgh, Edinburgh, United Kingdom; ^2^ Embodied AI Lab, Intel, Munich, Germany; ^3^ Department of Computer Science, University College London, London, United Kingdom

**Keywords:** reinforcement learning, legged robots, manipulation, robotics, robot learning, multi-expert learning, versatile locomotion

## Abstract

This work focuses on generating multiple coordinated motor skills for intelligent systems and studies a Multi-Expert Synthesis (MES) approach to achieve versatile robotic skills for locomotion and manipulation. MES embeds and uses expert skills to solve new composite tasks, and is able to synthesise and coordinate different and multiple skills smoothly. We proposed essential and effective design guidelines for training successful MES policies in simulation, which were deployed on both floating- and fixed-base robots. We formulated new algorithms to systematically determine task-relevant state variables for each individual experts which improved robustness and learning efficiency, and an explicit enforcement objective to diversify skills among different experts. The capabilities of MES policies were validated in both simulation and real experiments for locomotion and bi-manual manipulation. We demonstrated that the MES policies achieved robust locomotion on the quadruped ANYmal by fusing the gait recovery and trotting skills. For object manipulation, the MES policies learned to first reconfigure an object in an ungraspable pose and then grasp it through cooperative dual-arm manipulation.

## 1 Introduction

The re-usability of control policies is a challenging research area in robotics. Task-specific controllers and learned policies have been developed to achieve a wide range of tasks, such as locomotion (Bellicoso et al., 2018; Hwangbo et al., 2019) on quadrupeds, as well as manipulation of objects (Levine et al., 2018). To create useful versatile robots, the ability to perform multiple tasks is essential, however, combining and reusing these policies in a unified manner remains an open question. This is because a learned policy is trained specifically to solve a particular task which has limited applicability to transfer such a single-skilled policy to other tasks. Having a control policy applicable to other tasks commonly requires redesigning or retraining the policy.

This work investigates a systematic approach to formalise the synthesis of multiple learned policies—a multi-expert synthesis (MES) approach that can produce versatile robotic capabilities, in which a high-level behaviour policy recombines multiple skills and fuses more flexible ones, using specialised experts based on the observation of states and task. Here MES is defined as the process of combining or blending expert skills in a latent space. This will produce a multi-expert policy, where a high-level behaviour policy selects the appropriate experts according to the real-time state observation.

The essential principle of multi-expert synthesis lies in designing a hierarchical control architecture where non-relevant information is coordinated and hidden across the layers: while the high-level behaviour policy requires task-relevant information, e.g., distance to a goal or pose of the object to grasp, the low-level expert only requires the proprioceptive state of the robot. Thus, experts focus solely on performing their particular skills, while the high-level policy is responsible for drawing from the experts’ skills and completing the task.

This idea originates from Hierarchical Reinforcement Learning (HRL) (Barto and Mahadevan, 2003). In HRL, for discrete and tabular cases, temporal (Sutton et al., 1999) and state abstraction (Dietterich, 2000) determine the information that the components receive. Despite the advantages of encoding non-relevant information across layers, composing and synthesising expert knowledge is not possible in the standard HRL framework since only one expert can be selected at a time. This problem is addressed by learning continuous latent variables that blend the experts in a latent space (Haarnoja et al., 2018a; Merel et al., 2018b).

The traditional approach for the latent space blending is the Mixture of Experts (MoE), where the outputs of individual experts (actions) specialized on sub-problems are combined by a gating function (Jacobs et al., 1991). The core idea of MoE, combining the outputs of experts *via* a gating network, has been adapted in the areas of machine translation (Shazeer et al., 2017), computer vision (Chang et al., 2018), robotics (Mülling et al., 2013), Reinforcement Learning (RL) (Sun and Peterson, 1999), and Computer Graphics (Peng et al., 2019). However, MoE has limitations known as expert imbalance—a form of mode collapse that arises due to the lack of diversity across experts (Shazeer et al., 2017; Zhang et al., 2018).

Alternative to MoE, the Multi-Expert Learning Architecture (MELA) has been proposed, where the latent space is constructed by blending expert network weights leading to a higher-dimensional latent space representation of the multi-expert network than MoE (Yang et al., 2020b). It has been shown that MELA avoids the expert imbalance problem, and provides a framework to use experts for learning diversified skills as well as adaptive behaviours on a quadruped robot.

**FIGURE 1:**
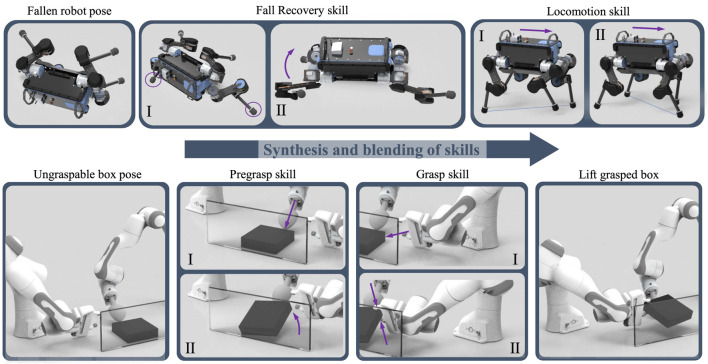
Synthesis of expert skills using Multi-Expert Synthesis. Top: Versatile locomotion by recovering from a prone position first and then transitioning into trotting. Bottom: Dual-arm cooperative manipulation, where the object is first reconfigured by the left hand into a graspable pose and then grasped by the right hand.

In this work, we present a systematic approach to best achieve MES and propose algorithms that address two key problems in multi-expert methods: 1) how to select the correct state space representation for the MES policies; and 2) how to avoid the lack of diversity across experts leading to expert imbalance. We will show the results of multi-expert policies for both quadruped locomotion and cooperative bi-manual manipulation (Figure 1).

Specifically in MES, where every expert and the gating network has an individual state space, to correctly design the state space is crucial and non-trivial: missing key state variables will prevent the policy from learning the right action; whereas having too many and unrelated states will result in slower learning, large variances in the reward signals, and higher probability of converging to local minima (Garcıa and Fernández, 2015).

Our proposed procedure addresses this problem by the automatic selection of task-relevant state variables. Traditionally, the state space is designed relying on domain knowledge, and extensive trial and error to find a state space representation that yields highest performance. However, with an increasing amount of experts for different skills this becomes time consuming and potentially infeasible. Our proposed automatic state space design process mitigates the aforementioned state space issues for MES, where multiple state spaces need to be designed for the individual experts and gating network.

In the context of MES, our proposed method to explicitly enforce diversity among experts allows multi-expert methods in general to not suffer from expert imbalance. In multi-expert systems, a common problem known as expert imbalance occurs. This is because when one expert is over-prioritised by the behaviour network, it results in downgrading of other experts and the inability to learn distinct expert behaviours.

Our contributions are summarised as follows:

1. A *systematic design for Multi-Expert Synthesis (MES)* enabling learning of skill coordination and effective synthesis of multiple experts during tasks.
2. Formulation of a *state selection algorithm* that uses the learned state value function to quantitatively identify essential states and rule out task-irrelevant variables.
3. A proposed *explicit enforcement of diversity among multiple experts* by maximising their discriminability.
4. Applicability of the proposed methods for MES to *achieve motor skill synthesis in both robot locomotion and manipulation.*

We validate the MES approach in two distinct domains of robotic skills: locomotion and manipulation. For the validation on a quadruped robot, we show that gait recovery and locomotion can be synthesised to achieve robust locomotion (Figure 1 Top). Furthermore, our method can be used for cooperative dual-arm manipulation (Figure 1 Bottom) to grasp an object from a previously ungraspable configuration. The effectiveness of the trained multi-expert policies were validated in a variety of test scenarios in both simulation and real-world experiments.

Following, we first present related work (Subsection 2). Next, we demonstrate how expert behaviour is obtained (Subsection 3). Next, we present MES, the state selection process, and the types of multi-expert policies in (Subsection 4). The results using MES for a quadruped and dual-arm, and a comparison between MoE and MELA is conducted in Section 5 and Section 6 respectively. Lastly, we conclude the work in Section 7.

## 2 Related work

Model-based control methods have been widely use for robotics tasks, such as locomotion, manipulation, and whole-body control (Siciliano and Khatib, 2016). In particular, recent optimisation based approaches enabled robots to plan (Winkler et al., 2018) and robustly realise planned motions (Kuindersma et al., 2016) under constraints. This paradigm of using optimisation to first plan and then robustly track the planned trajectory has found wide application in the control of floating-based robots (Feng et al., 2015; Johnson et al., 2015).

Despite a wide range of applications and problems solved through existing control methods, the requirement of large amounts of computation power for large-scale optimisation problems, dependence and necessity of accurate models, and their limitation to generalise under uncertainties remain a challenge. To this end, learning based methods offer an alternative paradigm by learning through data while only specifying high-level objects instead of detailed mathematical specifications of the physical environment. Beside optimisation methods, such as genetic algorithms (Mitchell, 1998), evolutionary optimisation (Ha and Liu, 2016), or Bayesian Optimisation (Snoek et al., 2012), Reinforcement Learning (RL) (Sutton and Barto, 2018) has been the main approach to solve problems which are difficult for classical control methods. While gradient-free optimisation methods have been mainly used to optimise for the parameters of a controller (Rai et al., 2018) or a model (Yuan et al., 2019), RL is learning a parametrised function representing both the control policy (Yang et al., 2020a) and the model (Sutton, 1990).

For complex tasks, such as continuous control in robotics, Deep Neural Networks have been used as function approximators for the actor and critic, opening the field of Deep Reinforcement Learning (DRL). In robotics, DRL has been used on problems that are hard to solve using classical control methods, such as control of life-like locomotion of humanoids (Yang et al., 2020a) and animals (Yang et al., 2020b), fall recovery for quadrupeds (Hwangbo et al., 2019), and hand-eye coordination for grasping (Levine et al., 2018).

Due to the data demanding nature of DRL algorithms, the policies are learned in simulation removing the risk of damaging the robot and leveraging advances in computation speed and parallel computing allowing fast collection of samples. To ensure that the simulation to reality (sim2real) transfer of the policy yields similar performances in reality as in simulation, the fidelity of the simulation can be increased (Tan et al., 2018; Hwangbo et al., 2019) or the policy can be trained to be robust to uncertainty (Peng et al., 2018b).

To complete multiple tasks using one unified framework, hierarchical control structures can be used to select appropriate sub-policies through a high-level gating mechanism. In HRL, complex tasks are solved by using existing knowledge of experts through temporal abstraction (Sutton et al., 1999). In its original form, temporal abstraction is achieved by having the top-level policy selecting among options, which are sub-policies that continuously perform one action over a period of time (Sutton et al., 1999).

With the advent of Deep Learning and Deep Neural Network, DRL has been used to extend the discrete tabular HRL concepts into the continuous control domain. In the context of multi-expert learning and multi policy composition, this led to frameworks where low-level experts encode motion primitives or skills, while a high-level policy selects the expert (Frans et al., 2017; Merel et al., 2018a). Alternative to learning a high-level policy to select appropriate motion primitives, COCoMoPL (Clever et al., 2017) proposes a framework, where near-optimal motion primitives are learned and synthesised into a motion as weighted combination of these motion primitives.

In (Kumar et al., 2018), a method is proposed that does not synthesise expert skills into a unified policy, but rather expands the existing skill by decomposing the task into simpler subtask and training a local policy for the subtasks.

A modular framework is proposed in (Lee et al., 2018), which learns transition policies that connect primitive skills to complete sequential tasks. An extended work in (Lee et al., 2019) uses the modular framework to learn to coordinate the learned primitive skills for task completion. In (Qureshi et al., 2019) a method is proposed that learns task-agnostic skills using their composite to solve new tasks in an HRL fashion.

In contrast to aforementioned methods that select one sub-policy, the outlined MES in this work continuously blends all experts. From a practical perspective, this allows the multi-expert policy to choose latent skills from multiple experts instead of just choosing a particular skill from one expert. Furthermore, MES continues to train the experts alongside the high-level behaviour network, such that the learned expert skills can be specialised and can learn new skills, while gating network blends the experts’ skills. To increase the usability and performance of MES, this work provides two novel algorithms for automatic state selection and enforcing expert diversity yielding higher performances and faster development times.

## 3 Generating expert behaviours

For the multi-expert learning structure, individual narrowly-skilled experts need to be obtained. In this work, two procedures are presented to obtain expert behaviours: 1) autonomously learning a policy through interactions with the environment and 2) learning to imitate a reference policy.

In order to solve a task, a reward, whose maximisation leads to task completion, needs to specified. If the policy is autonomously learning from scratch, methods from Deep Reinforcement Learning are deployed to maximise the reward. For imitation learning of a user-designed controller, a controller is designed first, and then a policy represented as Neural Network (NN) is trained to imitate the controller as closely as possible while maximising a task reward.

Using model-free DRL algorithms to learn a policy from scratch is helpful when the solution is not straightforward. On the other hand, using an existing manually designed controller gives more certainty of behaviours over the robustness, stability, and performance. Thus, combining control design for well-defined tasks, such as locomotion, and leveraging the exploration of learning methods for tasks in ill-defined or uncertain states and environments, such as fall recovery from any pose, provides experts for a variety of cases.

In the following, we will first introduce the control structure how the robot is controlled to achieve its task, and then present the procedure to learn experts through DRL. Lastly, we will show how an expert can be obtained from imitation learning.

### 3.1 Control structure

**FIGURE 2:**
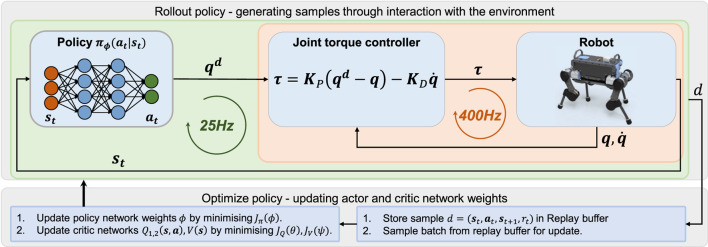
Learning framework to train specialised experts and multi-expert policies.

The control structure of the robot consists of two control loops: high-level behaviour control, and low-level joint impedance control (Figure 2). The high-level behaviour control loop governs the behaviour of the robot at 25 Hz by mapping the robot’s states into actions for the joint impedance controller, which runs at a frequency of 400 Hz. From measured joint positions and velocities, the joint torques are generated in a proportional-derivative control law:

$$\tau_{i}=K_{P}\left(q^{d}-q\right)-K_{D}\dot{q},$$ (1)

with joint index *i* = 1, …, *N*, joint torque *τ*, proportional gain *K*_*P*_, derivative gain *K*_*D*_, target joint position *q* ^*d*^, measured joint position *q*, and joint velocity $\dot{q}$ .

Here, a low-pass Butterworth filter is applied respectively on the feedback state and actions in the high-level control loop. Filtering the robot state allows reducing sensor noise while also providing smoother states for the Neural Network, thus yielding smoother target actions. Besides, to match the torque tracking bandwidth of the actuators, desired actions of the behaviours control are low-passed filtered and limited to those that are able to be executed by the actuators.

### 3.2 Training experts *via* deep reinforcement learning

To enhance the smoothness for realisable control actions on robotic systems, an additional smoothness objective is added to the standard maximum entropy objective (Ziebart et al., 2008) of SAC:

$$J\left(\pi \right)=J_{\text{SAC}}\left(\pi \right)-\lambda J_{\text{smoothing}}\left(\mu \right),$$ (2)

with stochastic policy *π*, smoothness regularisation parameter *λ*, deterministic policy *μ*, maximum entropy objective *J*_SAC_(*π*), and smoothness loss *J*_smoothing_(*μ*).

The maximum entropy objective *J*_SAC_(*π*) is defined as:

$$J_{\text{SAC}}\left(\pi \right)=\overset{T}{\underset{t=0}{\sum }}E\left[r\left(s_{t},a_{t}\right)+\alpha H\left(\pi \left(\cdot |s_{t}\right)\right)\right],$$ (3)

with state ***s***_*t*_, action ***a***_*t*_, temperature parameter *α*, expectation E of the reward *r* and policy entropy H(π) . The temperature parameter *α* governing the trade-off between exploration and exploitation is automatically adapted by minimising the objective *J*(*α*) (Haarnoja et al., 2018c):

$$J\left(\alpha \right)=E_{a_{t}∼\pi_{t}}\left[-\alpha \log\pi_{t}\left(a_{t}|s_{t}\right)-\alpha \bar{H}\right],$$ (4)

with minimum policy entropy threshold $\bar{H}$ .

The smoothing loss *J*_smoothing_(*μ*) encourages the policy to generate deterministic actions *μ*(***s***_*t*_) that are close to the current, measured state *q*:

$$J_{\text{smoothing}}\left(\mu \right)=\|\mu \left(s_{t}\right)-q{\|}_{2}.$$ (5)

Directly embedding a regularisation loss on the optimisation level yielded better smoothness, compared to the additional regularisation terms in the reward.

In the SAC algorithm, the parameters of three function approximators are learned: parameters *ϕ* of the policy *π*_*ϕ*_, parameters *θ* of the soft action-value function *Q*_*θ*_, and parameters *ψ* of the soft state-value function *V*_*ψ*_.

After applying the reparametrization trick, the policy parameters *ϕ* can be learned by minimising the objective *J*_*π*_(*ϕ*) (Haarnoja et al., 2018b):

$$J_{\pi }\left(\phi \right)=E\left[\log\pi_{\phi }\left(a_{t}|s_{t}\right)-Q_{\theta }\left(s_{t},a_{t}\right)\right]+J_{\text{smoothing}}\left(\mu_{\phi^{1}}\right).$$ (6)

The policy *π*_*ϕ*_ is re-parametrised through a neural network transformation: the action $a_{t}=f\left(\mu_{\phi^{1}},\sigma_{\phi^{2}}\right)$ is sampled from a squashed Gaussian distribution $f\left(\mu_{\phi^{1}},\sigma_{\phi^{2}}\right)=\tanh\left(N\left(\mu_{\phi^{1}},\sigma_{\phi^{2}}\right)\right)$ . The deterministic policy $\mu_{\phi^{1}}$ and the standard deviation $\sigma_{\phi^{2}}$ are Neural Networks parametrised by the weights *ϕ* ^1^ and *ϕ* ^2^ respectively.

For stability of the training, the more conservative estimation between two Q networks $Q_{\theta_{1}}$ and $Q_{\theta_{2}}$ is used for the action value function *Q*_*θ*_(***s***_*t*_, ***a***_*t*_):

$$Q_{\theta }\left(s_{t},a_{t}\right)=\min_{j=1,2}Q_{\theta_{j}}\left(s_{t},a_{t}\right).$$ (7)

Minimising Bellman residual *J*_*Q*_ (*θ*_*j*_) with bellman equation $\hat{Q}\left(s_{t},a_{t}\right)=r\left(s_{t},a_{t}\right)+\gamma E\left[V_{\bar{\psi }}\left(s_{t+1}\right)\right]$ and discount factor *γ* yields the parameters *θ*_*j*_ for action-value function $Q_{\theta_{j}}\left(s_{t},a_{t}\right)$ :

$$J_{Q}\left(\theta_{j}\right)=E\left[\frac{1}{2}{\left(Q_{\theta_{j}}\left(s_{t},a_{t}\right)-\hat{Q}\left(s_{t},a_{t}\right)\right)}^{2}\right].$$ (8)

The parameters $\bar{\psi }$ of the target value function network $V_{\bar{\psi }}\left(s_{t+1}\right)$ are obtained by polyak averaging the parameters *ψ* through minimising the objective *J*_*V*_(*ψ*):

$$J_{V}\left(\psi \right)=E\left[\frac{1}{2}{\left(V_{\psi }\left(s_{t}\right)-E\left[Q_{\theta }\left(s_{t},a_{t}\right)-\log\pi_{\phi }\left(a_{t}|s_{t}\right)\right]\right)}^{2}\right].$$ (9)

#### 3.2.1 State observation

Correctly designing state space S with only essential state variables provides clear reward signals and better success rate of learning good policies. On the contrary, using irrelevant states introduces large variances in the reward or feedback signals, and consequently decrease performance. Furthermore, from a computational perspective, high-dimensional state spaces require more data to generalise the whole state space, and impede convergence because the non-linear function approximator is not able to inter- or extrapolate the high-dimensional state space due to the curse of dimensionality.

**FIGURE 3:**
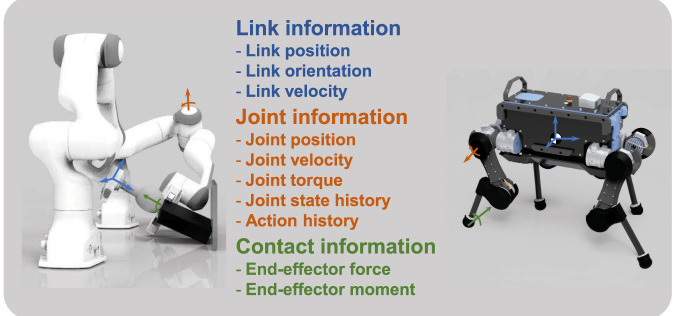
Potential state variables as feedback states for locomotion and manipulation tasks.

In Section 4.2 of the main manuscript, we proposed a systematic approach to choose the correct subset of states among all possible variables. Figure 3 depicts a non-exhaustive set of potential states for floating-base and fixed-base systems, such as quadrupeds and manipulators respectively.

For all physically measured quantities, a first-order low-pass Butterworth filter is applied to denoise. The cut-off frequency is individually set and is determined through a spectral analysis based on signals from the idle system.

#### 3.2.2 Action space

For the choice of the action space A , two established options are considered: *joint space* for controlling the joints and *task space* for controlling the end-effector pose. In this work, we use the joint positions for quadrupeds and end-effector poses for manipulators.

#### 3.2.3 Reward design

As the reward function governs the behaviour of the policy, ambiguously specifying the reward could lead to reward exploitation and potentially failures. Here, we formulate a set of physically-motivated reward terms *r*_*i*_ for both floating-based and fixed-based systems.

The reward *r*_*t*_ at time *t* is calculated as weighted sum over the individual reward terms *r*_*i*_ with weights *w*_*i*_:

$$r_{t}=\overset{N}{\underset{i=0}{\sum }}w_{i}r_{i}.$$ (10)

To both regularise and achieve desired values, a Gaussian Radial Basis Function (RBF) $r_{i}:R\to \left(0,1\right]$ is used:

$$r_{i}\left(x\right)=\exp\left(-\kappa \|x_{d}-x{\|}^{2}\right),$$ (11)

with desired value *x*_*d*_ and current value *x*. For regularisation terms, the desired value *x*_*d*_ is set around the operating point. The width *κ* governs the tolerance *δ*_max_ of the residual error, and is calculated as $\kappa =-\text{ln}\left(C\right)/\delta_{\max}^{2}$ with small associated reward *C≐*0.001 at the boundaries of the tolerance. We use an approximation *C* → 0 since ln (*C* = 0) is infinite.

Although there is no general rule for reward shaping—the process of adding and removing reward terms and tuning the relative weights between each other—the following guidelines can be considered and proved helpful during the process of designing policies for locomotion, gait recovery, pregrasping, and grasping on quadrupeds and dual arm manipulators respectively.

• High weights on goal related terms, e.g., small residual error between joint angles or poses for imitation terms, small distance to goal (grasping), or small residual error in body velocities (locomotion);
• Low weights on regularisation terms of physically meaningful terms. Regularisation on quantities, such as joint torque or joint velocity, or regularisation around nominal positions, proved helpful for stability, reducing and oscillation. This behaviour is similar to regularisation for stability and robustness purposes in optimisation in Robotics, e.g., Whole-Body Optimisation Yuan et al. (2019) or Trajectory Optimisation Winkler et al. (2018).
• The usage of reward terms that are non-negative or positive terms instead of negative terms should be chosen. Having negative rewards terms encourages the agent to terminate early in order to prevent culminating more negative rewards. Radial Basis Functions were found suitable as kernel and provides good gradients. Other non-zero kernels are suitable as well.

All single-task experts and multi-expert policies in this work are trained by maximising rewards to follow desired link poses, and regulate joint velocity and joint torque for smoothness in actions. The rewards used in this work can be found in Section 5.3 of the main manuscript, and a more detailed description for reward design can be found in (Yang et al., 2020a, 2018; Hwangbo et al., 2019; Sun et al., 2020).

#### 3.2.4 Training procedures

Designing training procedures for the DRL agent is required for both the success and high quality policies. We applied four techniques during the training of the policy: early termination, reference state initialisation (RSI), guided curriculum, and dynamics randomisation.

Early termination stops an episode when meeting an early termination criterion and is therefore used to discard irrelevant and skewed samples. Early termination biases the policy to avoid bad states as the agent cannot collect any further rewards if the episode is terminated. Early termination criteria are set for states that have self-collision or unwanted collisions with the environment. For floating-base systems, once the height of the base falls below a threshold, the episode is terminated.

In order to generalise well across the whole state space and thus training a robust policy, the robot is initialised in reference states that are task-relevant but rarely encountered (Peng et al., 2018a). Reference states include failure states, local minimum solutions, or imitation frames (see Section 3.3).

For best performance of the policy, curriculum for the learning process is applied. The curriculum aims to guide the policy by gradually increasing the difficulty of the task. In general, if the task difficulty is too high, the policy converges to a locally optimal solution. An increase in the difficulty can be implemented on reward weight, e.g., increasing importance on regularisation weights (Hwangbo et al., 2019), or in the amount of tasks which the policy needs to complete, e.g., initially standing for a quadruped robot and gradually adding locomotion tasks while withstanding disturbances or an increase in dynamics randomisation (Yang et al., 2018). In our setting, the robot is being pushed during training as in (Yang et al., 2018) to provide additional robustness towards uncertainties.

To increase the robustness of the policy against model uncertainties and to improve the transferability of policies across environments, we applied dynamics randomisation (Peng et al., 2018b) on the robot model. At the beginning of every episode, the parameters of the robot dynamics are uniformly randomly sampled within the range specified in Table 1 in the Supplementary Materials.

**TABLE 1:** Variations of dynamics randomisation for training.

	Default value	Min (training)	Max (training)	Min (testing)	Max (testing)
Contact friction	0.7	50%	150%	30%	170%
Joint torque	40Nm	80%	120%	80%	200%
Nertia	link dependent	80%	120%	50%	150%
Mass	link dependent	80%	120%	50%	150%

### 3.3 Training expert *via* imitation learning

As an alternative to learning *via* DRL from scratch, we use imitation learning to train a DRL agent by imitating a reference trajectory. In the following, we present a learning scheme for quadruped locomotion that encourages the agent to imitate a reference motion while solving a task as described in Section 3.2.3.

Four quantities are used for the learning algorithm to imitate: joint positions, joint velocities, relative end-effector positions, and contact states of the feet. The references are time-based trajectories obtained from an optimal trajectory generator for the Centre of Mass (CoM) and the whole-body controller that tracks the CoM trajectory (Bellicoso et al., 2017; Yuan and Li, 2018). For imitation learning, the reward is modified. The new reward consists of a task reward *r*_task_, and an imitation reward *r*_imitation_:

$$r_{t}=w_{\text{task}}r_{\text{task}}+w_{\text{imitation}}r_{\text{imitation}},$$ (12)

with weights *w*_task_, *w*_imitation_ corresponding to the importance of task completion and imitation quality respectively.

The imitation reward *r*_imitation_ is calculated as the weighted sum of four sub-rewards:

$$r_{\text{imitation}}=w_{q}r_{q}+w_{\dot{q}}r_{\dot{q}}+w_{\text{eef}}r_{\text{eef}}+w_{\text{contact}}r_{\text{contact}}.$$ (13)

The joint position *r*_*q*_, joint velocity $r_{\dot{q}}$ , and end-effector position *r*_eef_ rewards are formulated as Gaussian RBF encouraging similarity between demonstrated and actual robot state. For the contact state, a reward of 1 is assigned if all four contact states match the demonstrated contact state, and is 0 otherwise.

To prevent temporal ambiguity during the imitation of a time series of reference frames, a variable representing time needs to be provided, similar to (Peng et al., 2018a; Yang et al., 2020a). Because of the periodic nature of locomotion, a periodic phase vector ***ζ*** is formulated as:

$$\zeta =\left[\begin{array}{c} \sin\left(\Omega t\right)\\ \cos\left(\Omega t\right) \end{array}\right],$$ (14)

where Ω normalises the period to match the periodicity of the reference time series, and the representation using ***ζ*** is compatible with NN instead of using a monotonically increasing time as a variable.

For RSI, the robot’s joint position and joint velocities are initialised according to a uniformly random sample from the reference trajectory.

## 4 Multi-expert synthesis

In the following, we outline Multi-Expert Synthesis (MES) and elaborate how the trained experts are embedded in MES. We further formulate an automatic State Space Selection Process that was used to determine the state space of all policy networks. Lastly, we present how to enforce diversity among experts and tackle the problem of mode-collapse.

### 4.1 Learning structure

**FIGURE 4:**
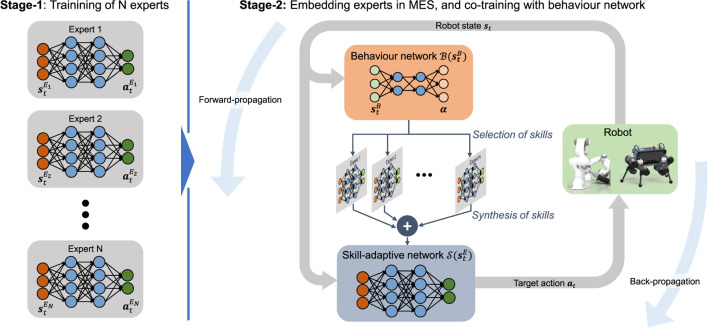
Multi-Expert Synthesis trained in a two-stage curriculum. Experts are pre-trained in Stage-1 and co-trained in Stage-2 alongside a Behaviour Network.

The two-stage training process for MES policies is depicted in Figure 4. In the first stage, individual, single-skilled experts are trained by solving a particular task as described in Section 3. In the second stage, the high-level behaviour network is trained alongside the pre-trained experts. While being synthesised by the high-level behaviour network, the pre-trained experts in the second stage are further fine trained which allow all experts to synergise with one another.

Based on the behaviour state $s_{t}^{B}$ , the behaviour network $B\left(s_{t}^{B}\right),B:R^{\text{dim}\left(s_{t}^{B}\right)}\to R^{N}$ continuously synthesises the *N* task-specific expert skills and builds a skill-adaptive network $S\left(s_{t}^{E}\right)$ . The skill-adaptive network (blue shaded network in Figure 4) infers actions $a_{t}=S\left(s_{t}^{E}\right)$ from expert state $s_{t}^{E}$ .

For this work, we consider two approaches—MELA and MoE—to achieve MES. The difference between MELA and MoE lies in how the outputs $\alpha =B\left(s_{t}^{B}\right)$ , $\sum_{i=1}^{N}\alpha_{i}=1$ from behaviour network *B* are used as weights to synthesise the expert networks into the skill-adaptive network.

For MELA, the outputs of the behaviour network ***α*** blend the network parameters of the experts (Yang et al., 2020b), while MoE ***α*** uses the weighted sum of the actions ***a***_*i*_ of all expert networks.

#### 4.1.1 Multi-expert learning architecture

The skill-adaptive network’s parameters $\theta_{n,\text{SAN}}^{\left[l\right]}$ are obtained as the weighted sum of the expert’s parameters $\theta_{n,i}^{\left[l\right]}$ :

$$\theta_{n,\text{SAN}}^{\left[l\right]}=\overset{N}{\underset{i=1}{\sum }}\alpha_{i}\theta_{n,i}^{\left[l\right]},$$ (15)

for the *i*-th expert, neuron *n* in layer *l*, and blending weights $\alpha =B\left(s_{t}^{B}\right)$ with $\sum_{i=1}^{N}\alpha_{i}=1$ from the behaviour network *B*. All operations in the architecture are differentiable that allow backpropagation, and the networks are trained with SAC.

**FIGURE 5:**
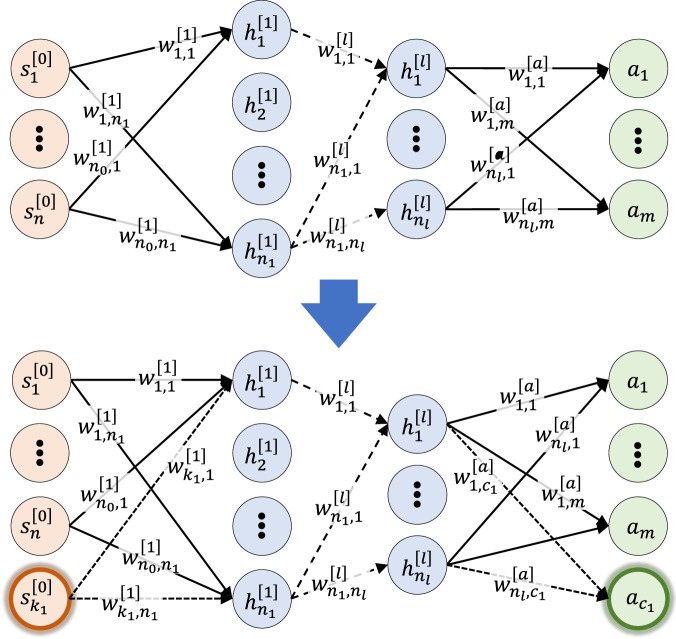
Augmented expert network. Top: Originally trained expert. Bottom: Augmented expert network. All neurons are fully connected including bias terms but omitted for clarity.

As the skill-adaptive network’s parameters are a linear combination of all expert parameters, which have the same amount of neurons and layers. Thus, to accommodate different expert network sizes, the expert network size in Stage-2 is augmented (Figure 5). The amount of layers and neuron per layers are the same as the largest network. To keep the input-output behaviour of the augmented neural network unchanged, all weights $w_{k,j}^{\left[l\right]}$ of the newly added neuron connections are initialised to zero. The output of the *j*-th neuron $h_{j}^{\left[l\right]}$ thus remains unchanged for $w_{k,j}^{\left[l\right]}=0$ :

$$h_{j}^{\left[l\right]}=f^{\left[l\right]}\left(\overset{n}{\underset{i=0}{\sum }}w_{i,j}^{\left[l\right]}x_{i}^{\left[l-1\right]}+\overset{n_{k}}{\underset{k=n+1}{\sum }}w_{k,j}^{\left[l\right]}x_{i}^{\left[l-1\right]}+b_{j}^{\left[l\right]}\right),$$ (16)

with *n* neurons in layer *l*, activation function *f* (⋅), weight $w_{i,j}^{\left[l\right]}$ , output $x_{i}^{\left[l-1\right]}$ of the previous layer, and bias *b*. Examples for network augmentation of locomotion and manipulation policies can be found in Section 5.3 (Figure 5).

#### 4.1.2 Mixture of experts

For MoE, the actions ***a*** are calculated as weighted sum from the expert’s actions ***a***_*i*_ and the behaviour network’s output $\alpha =B\left(s_{t}^{B}\right)$ :

$$a=\overset{N}{\underset{i=1}{\sum }}\alpha_{i}a_{i},$$ (17)

where *N* is the number of experts with corresponding weights *α*_*i*_ on the output actions ***a***_*i*_. Consequently, compared to MELA, the dimensionality of the latent space of MoE is much lower by orders of magnitudes.

### 4.2 Automatic state space selection

In the following, we present the systematic selection of the state space based on their relevance to the task. The core idea of the state selection process is to check whether the removal of a sub-state space $S^{-}$ from state space $S^{*}$ has an influence on the value function V(s),∀s∈S .

The value function $V:R^{n}\to R$ describes the accumulative reward gained in an episode by starting in state ***s*** and applying policy *π*(***a*** ∣ ***s***) successively. If the reduced state space $S^{+}{=S}^{*}{\backslash S}^{-}$ yields a value *V* (***s*** ^+^) similar to the complete *V* (***s*** *), then $S^{-}$ has no influence on the accumulated reward, and can be thus omitted.

More specifically, we have:

$$\begin{array}{l} V\left(s^{+}\right)≃V\left(s^{*}\right),\forall s^{+}{\in S}^{+},s^{*}{\in S}^{*},\\ \Leftrightarrow \\ S^{-}\text{is not required}\text{ for the task}, \end{array}$$ (18)

where $S^{+}{\cup S}^{-}{=S}^{*}{,S}^{+}{\cap S}^{-}=\emptyset$ .

To describe the various state quantities, we use the following notation. Symbols written in bold denote a state space $S\in R^{n}$ with *n* dimensions. The *k*-th dimension of the state space $S={⋃}_{k=1}^{n}S_{k}$ is denoted as $S_{k}\in R\subset S$ , and its members are called state variables $s_{k}\in S_{k}$ . In the context of state space reduction, we use state space $S^{*}={⋃}_{k=1}^{n}S_{k}$ as the union of relevant $S^{+}$ and irrelevant $S^{-}$ sub-state spaces. A vector s∈S is called a state.

In Algorithm 1, we outline how the state space is selected. Algorithm 2 describes the practical implementation of (18) to determine relevant state variables. For selecting relevant sub-state spaces (Algorithm 1), the state space is repeatedly reduced using Algorithm 2. During every iteration, value function *V* ^*π*^(***s***) and policy *π*(***a*** ∣ ***s***) are trained (Section 3) using states $s{\in S}^{i}$ . If the state space is not further reduced, the task-relevant state space $S^{+}$ is found. Otherwise, a new policy and value function is trained using the newly reduced state space $S^{i+1}$ .

In our work, the task-relevant state space is usually found after one iteration. However, when no domain knowledge for the initial state space $S_{0}$ can be applied, then all possible state dimensions described in Section 3.2.1 of the Supplementary Materials are used and more iterations are needed. This is because the learned value function *V*(***s***) is not capable of approximating a high-dimensional state’s value with the given amount of data. In this case, the state space needs to be successively reduced (line 5 in Algorithm 1) for a better approximation of the state’s values.


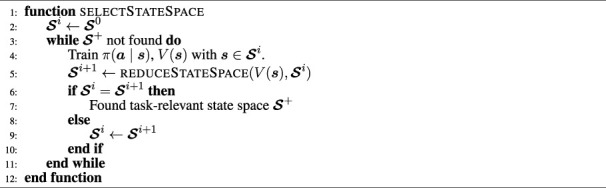

Algorithm 1 Pseudo code for relevant state selection.

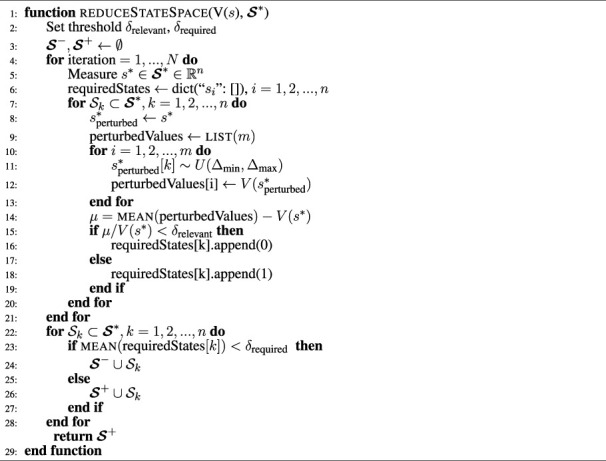

Algorithm 2 Pseudo code for selecting relevant state variables.

For the reduction of the state space in Algorithm 2, two thresholds *δ*_relevant_, *δ*_required_ are set. The *relevance* of sub-state space $S_{k}$ is evaluated *N* times. If the average relevance exceeds threshold *δ*_required_, then sub-state space $S_{k}$ is *required*. Averaging the relevance is necessary due to the variance in the estimation of the value function *V*(*s*).

At every iteration of a state’s relevance (line 4 in Algorithm 2), the relevance of every sub-state space $S_{k}$ is evaluated by perturbing the corresponding state variable *s*_*k*_ of a measured state ***s***. The state ***s*** is measured while rolling out policy *π*(***a*** ∣ ***s***). The state variable *s*_*k*_ is perturbed *m* times by setting their values to a uniformly sampled value within [Δ_min_, Δ_max_] covering the sub-state space $S_{k}$ for *m* → *∞*.

If the percental change of the average perturbed value is small for state variable *s*_*k*_, the sub-state space $S_{k}$ is considered irrelevant to the cumulative reward. If the relevance averaged over *N* times is below threshold *δ*_required_, implying that the sub-state $S_{k}$ is irrelevant across the whole state space $S^{*}$ for *N* → *∞*, we determine sub-state $S_{k}$ as not required.

In MES, state selection (Algorithm 1) is conducted for every expert during Stage-1 and for the behaviour network in Stage-2. Network augmentation [Eq. 16] is used in Stage-2 to unify the network sizes across the experts.

### 4.3 Expert diversification

In multi-expert systems, expert imbalance commonly occurs. For tasks requiring multiple experts as shown in Section 5.4, expert imbalance leads to the task not being completed and a local minimum solution.

To prevent expert imbalance, we encourage the experts to learn easy-to-discriminate and diverse skills by including a discriminator objective (Eysenbach et al., 2018) in the DRL objective (see Eq. 2):

$$J_{\text{diversity}}\left(q_{\phi }\right)=\log q_{\phi }\left(z|s^{D}\right),$$ (19)

with learned discriminator *q*_*ϕ*_(*z*∣ ***s*** ^*D*^) parametrised by weights *ϕ* and one-hot vector *z* indicating the skill. The discriminator estimates the likelihood of skill *z* conditioned on state ***s*** ^*D*^, and is trained to minimise the cross entropy $H\left(\hat{z},z\right)$ between real skill $\hat{z}$ and predicted skill *z*:

$$J_{\text{discriminator}}=\frac{1}{N}\overset{N}{\underset{i=1}{\sum }}H\left({\hat{z}}_{i},z_{i}\right).$$ (20)

The diversity objective 19) encourages the policy to produce states as distinct as possible, so that the discriminator can easily estimate the skill *z* based on the state ***s*** ^*D*^. Note that the state $s^{D}{\in S}^{D}$ can different from the expert state space $S^{E}$ . The discriminator state space $S^{D}\in R^{6}$ used for the locomotion discriminator uses orientation and velocity to discriminate between fall and locomotion. Beside explicitly enforcing skill diversification, it shall be noted that implicit diversity naturally emerges in MELA and RSI (Yang et al., 2020b).

## 5 Results

In this section, first we outline the setup of the learning framework. Second, we present results of our proposed state selection process. Third, the expert behaviours of MES are shown. Next, Multi-Expert results on the quadruped and dual-arm robot are reported. Lastly, we show the robustness and ability of MES to generalise across environments.

We demonstrate that our state selection process finds the state space yielding the highest reward and is used to design the state space for all MES components. Furthermore, MES learns robust multi-expert policies that are effective from simulation to the real robots and in unseen test scenarios.

### 5.1 Training setup

All policies were trained in PyBullet (Coumans and Bai, 2016) on a commercial computer (CPU: i7-7700K, GPU: Nvidia GTX 1080Ti). The expert and multi-expert policies converged after 1000 and 2000 (Figure 13) epochs respectively. Every epoch consisted of 1000 samples using the sample collection depicted in Figure 2 with 25 samples collected per second.

All networks are two-layered, fully connected Neural Networks with 256 neurons in each layer. Rectified Linear Units (ReLU) were used as activation functions. The standard parameters of SAC were used as in (Haarnoja et al., 2018b).

We performed dynamics randomisation (Peng et al., 2018b) during the training (see Section 3.2.4) and transferred the policy from PyBullet to both Gazebo (Koenig and Howard, 2004) and the real robots. During training in PyBullet a range of values were used for dynamics randomisation (see Table 1 training in Supplementary Materials), and a larger range (see Table 1 testing in Supplementary Materials) was used in Gazebo, which show that the multi-expert policy is robust in presence of large physical discrepancies.

### 5.2 Comparison of different state observations

The state selection process described in Section 4.2 was used to design the state space for the experts and gating network for all policies. We demonstrate the state selection process and its effectiveness on quadruped locomotion and reach-grasp manipulation (see Section 5.3).

First, we determine the relevant state variables from a large set of potential state variables. Second, we show that our proposed method distinguishes between completely irrelevant state variables and potentially relevant state variables by adding 10 random variables to the state space. Lastly, we compare the performance of optimally selected state spaces and extended state space containing irrelevant state information based on quantitative performance and qualitative behaviour.

We chose and defined the state variables for initial state space $S_{0}$ as: linear base velocity $\dot{x}$ , angular base velocity *ω*, gravity vector *g*, phase *p*, joint position *q*, joint velocity $\dot{q}$ , joint torque *τ*, end-effector force *f*_*c*_ for locomotion, and additionally end-effector position *x*_*eef*_ and velocity ${\dot{x}}_{\mathrm{eef}}$ , and quaternion $Q_{\mathrm{eef}}$ for manipulation.

**FIGURE 6:**
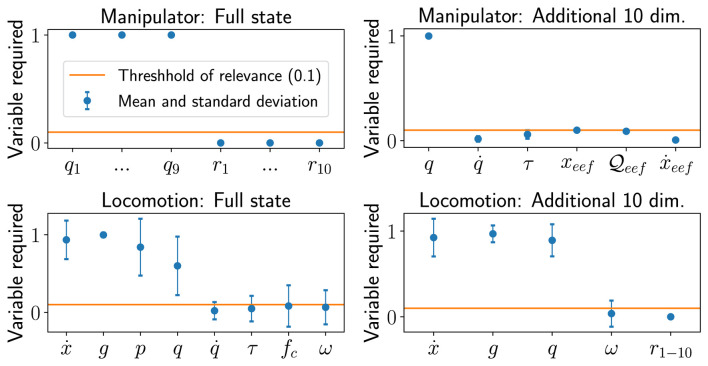
Results of state selection using large state space and 10 additional dimensions for locomotion and manipulation. Top left: Full state variables for manipulation, top right: additional 10 values as state variables, bottom left: full state variables for locomotion, bottom right: additional 10 values as state variables for locomotion.

After one iteration of Algorithm 1 with threshold *δ*_required_ = 0.1, the least relevant state variables were found to be: joint velocity and torque, and end-effector force for locomotion (Figure 6 top left) and all state variables but the joint positions for manipulation (Figure 6 bottom left). By removing the least relevant state variables from the initial, extended state space $S_{0}$ , we obtain a reduced state space with only task-relevant state variables.

The state selection process was further validated by adding 10 random variables *r*_*i*_, *i* = 1, …, 10 to the reduced state space. For every sample, the value of every random variable *s*_extra,*i*_ was uniformly sampled *s*_extra,*i*_ ∼ *U* ( −1, 1). All 10 random variables (Figure 6 top and bottom right) were identified to be irrelevant after one iteration of Algorithm 1.

Here, we define our formulation of reduced state space as the baseline, and those with all possible states as the extended state space including those irrelevant to the task, as found by our algorithms, for both locomotion and manipulation. We comparatively analysed the difference between the *baseline* and the *extended* state space representations in terms of the learning curves and the learned behaviours.

**FIGURE 7:**
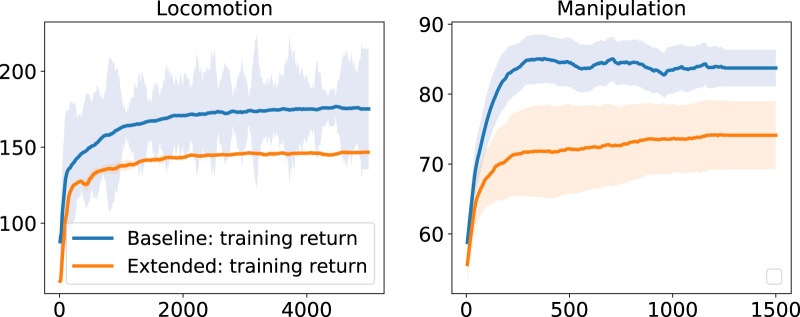
Performance for different state space configurations for locomotion (left) and manipulation (right).

Our baseline state space converged to higher rewards in both locomotion and manipulation tasks (Figure 7). In contrast, the extended state space was easily stuck in local minima in almost 60% of the cases, leading to policies with lower return and the incompletion of the task. For a fair comparison, the local minima solutions that did not complete the task were not included in the learning curves.

**FIGURE 8:**
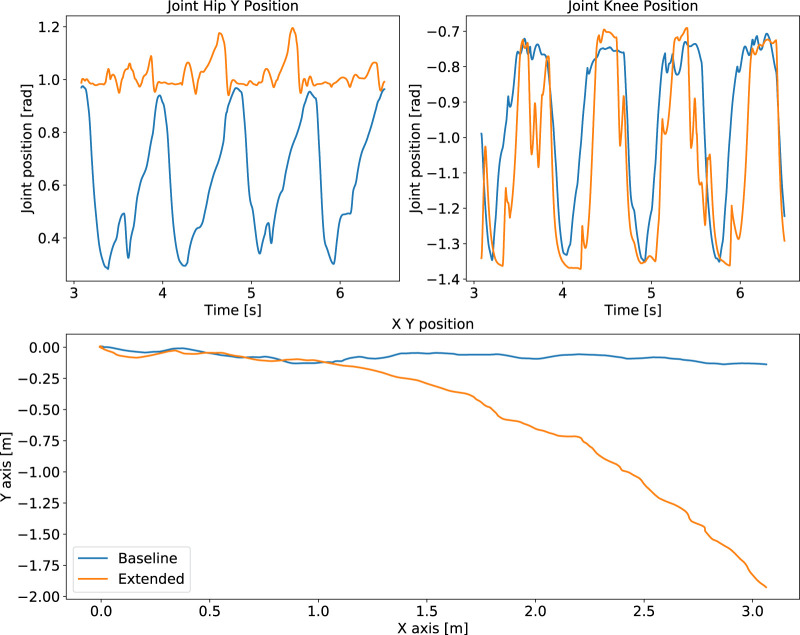
Locomotion performance: comparison of policies trained using the optimal baseline state space versus the extended states. Failure to capture periodicity (top) and drift of robot position (bottom) from the extended state space policy (orange), compared to our formulated baseline policy with optimal state space (blue).

**FIGURE 9:**
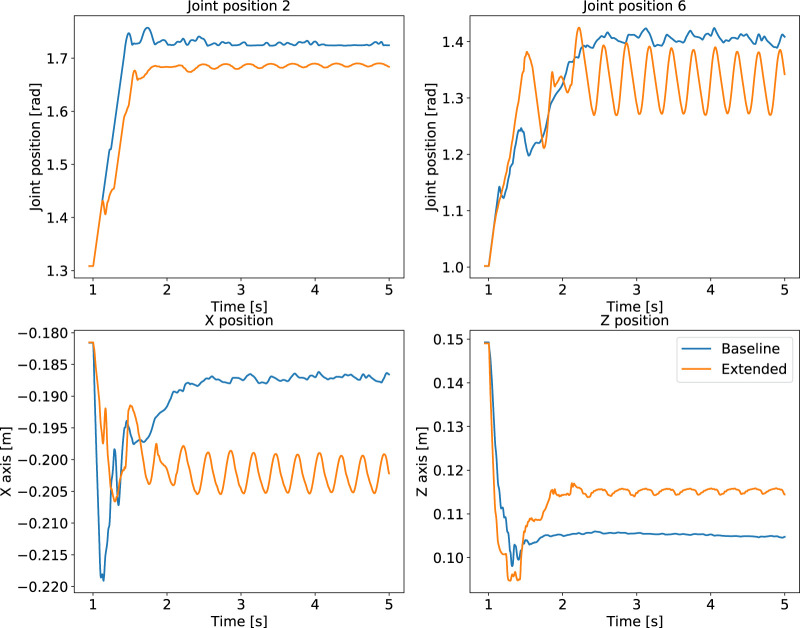
Manipulation performance: comparison of policies trained using the optimal baseline state space versus the extended states for. Oscillatory behaviour using the extended state space (orange), compared to our formulated baseline policy (blue).

Another difference in performance between baseline and extended state space is observed from the policy’s behaviours. While the extended state space representation completes the tasks, our proposed baseline performs better than the extended case. During quadruped locomotion the extended state space representation drifts in the *y* direction and is not able to encode the periodicity of locomotion (Figure 8). Besides, the manipulation policy exhibits oscillatory behaviour after grasping the object (Figure 9).

### 5.3 Expert behaviours

**FIGURE 10:**
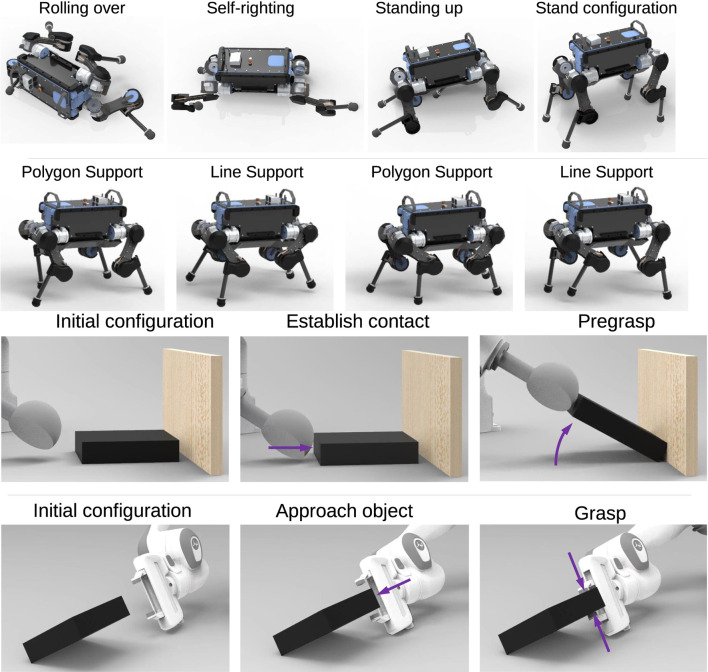
Expert policies for gait recovery (first row), trotting (second row), pregrasping (third row) and grasping (fourth row).

Using the training procedure in Section 3, all experts were trained for the use in Stage-2 of MES (Figure 4). In Figure 10, two expert behaviours are shown: gait recovery and trotting. Please see the accompanying video for further details.

#### 5.3.1 Locomotion experts

The gait recovery and trotting experts were trained as follows. The training of the gait recovery expert was conducted through directly specifying a reward, with which the maximisation leads to task completion. For trotting additional reference trajectories were provided for imitation (see Section 3.3). The weights of the reward terms can be found in Table 2 of the Supplementary Materials.

**TABLE 2:** Reward weights for quadruped experts.

	*w*task	*x*vel	*y*vel	*z*vel	*z*pos	g^*L*^	reg	*w*_imit:_	*q*	*q*˙	cont.	Eef pos
r_gr_	1.0	1	1	1	5	10	1					
r_stand_	1.0	2	2	2	4	4	1					
r_loco_	0.3	6	1	0	1	3	1	0.7	0.5	0.2	0.05	0.25

All reward terms were expressed as radial basis function (see Section 3.2.3) unless stated otherwise. For gait recovery and push recovery, the sagittal velocity *x*_vel_ was set to zero; for the locomotion gaits, *x*_vel_ was provided by the imitation data, and the lateral and vertical velocities *y*_vel_, *z*_vel_ were zero. The unit gravity vector *g* ^*L*^ is the normalised gravity vector in the robot body frame. Regularisation was performed on the joint velocities and joint torques by using a zero vector as target value. For the imitation terms, the target values were provided by the imitation data from a trotting controller.

Early termination was conducted for all experts if the robot was in self-collision. For locomotion, the episode terminated early if any link but the feet was in contact with the ground or if the body height fell below 0.25 m. RSI was performed for both locomotion and gait recovery. For locomotion, the robot was spawned in joint states from the reference imitation data. For gait recovery, the robot was spawned in prone and supine body poses with random joint positions.

#### 5.3.2 Manipulation experts

For dual-arm cooperation, pregrasping and grasping experts were trained (Figure 10). The state space of both manipulators were determined using Algorithm 1. For grasping, the state space $S_{\text{grasp}}\in R^{9}$ consists of 9 joint positions of the manipulator. The action space consists of the end-effector’s pose and the parallel grasper’s joint position $A_{\text{grasp}}\in R^{8}$ . The pregrasp expert uses end-effector pose as action space $A_{\text{grasp}}\in R^{6}$ , and 7 joint positions and the object pitch angle for state space $S_{\text{pregrasp}}\in R^{8}$ . Both experts use a two-layered neural network with 256 neurons in each layer.

For pregrasping, the reward has a contact and object orientation term with weights *w*_contact_ = 1 and *w*_object_ = 3 respectively. A reward of 1 was assigned when the end-effector was in contact with the object. The residual error in (11) was calculated as max (*θ*_*d*_ − *θ*, 0) with desired orientation *θ*_*d*_ = 45°.

The grasping reward is the sum of finger contact and end-effector position reward with weights *w*_contact_ = 1 and *w*_eef_ = 1 respectively. A reward of 1 was assigned if both fingers were in contact with the object. A reward for the end-effector link being close to the desired position ***p***_*d*_ was assigned (Eq. 11). Early termination was performed in case of self-collision or if any link other than the end-effector was in contact with the object.

### 5.4 Multi-expert results

The previously learned expert skills are now used for MES to achieve robust locomotion and dual-arm manipulation. We choose MELA over MOE as the multi-expert framework because MELA yields better policy performance during domain transfer and is able to diversify experts better (see Section 6).

#### 5.4.1 Robust locomotion

Two experts per skill were initialised for further diversification. Using the network augmentation shown in Figure 5, the expert’s state space can be preserved while being embedded in Stage-2 of MES. Early termination was applied in case of self-collision. The pose for RSI was uniformly sampled between prone positions and reference imitation trajectories.

**FIGURE 11:**
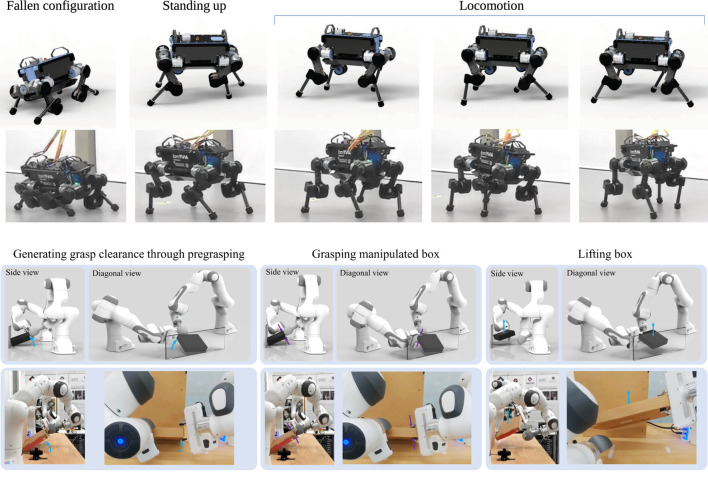
Multi-expert policies in simulation and on the real robot. Top: Robust locomotion of gait recovery from a prone position and smooth transitions to trotting. Bottom: Dual-arm cooperative manipulation and grasping.

For robust locomotion, the gait recovery expert reward *r*_gr_ is used if the robot falls, i.e., the threshold in height *p*_*z*_ < 0.4*m* or body orientation *rpy* > 20° is exceeded; and the locomotion expert’s reward *r*_loco_ is used, otherwise. For the behaviour network’s state space, the state selection process results in sufficient forward velocity. The motion can be seen in Figure 11 and in the accompanying video.

#### 5.4.2 Dual-arm cooperation

The dual-arm manipulation results show how MES adapts the experts’ access to information using the network augmentation technique depicted in Figure 5. For the pregrasping expert, although the object’s orientation is relevant during the Stage-1 training, it became less relevant after co-training with a behaviour network in Stage-2. The relevance of the object’s orientation was determined by looking at the values of the weights and gradient related to the object orientation, which were almost zero. From the hierarchical structure, this is explainable since the behaviour network can access the object’s orientation and thus activate the expert accordingly.

We found that MES consistently adapted the experts’ requirement of state variables throughout all trained policies. The expert policies for the pregrasping and grasping task only used joint positions of the pregrasper and grasper respectively, while the behaviour network used the object’s orientation as state input. Backpropagation through the MES network allowed the experts and behaviour network to share the state information for completing the task. The coordinated motions can be seen in the bottom of Figure 11 and the accompanying video.

### 5.5 Robustness and versatility of multi-expert synthesis

**FIGURE 12:**
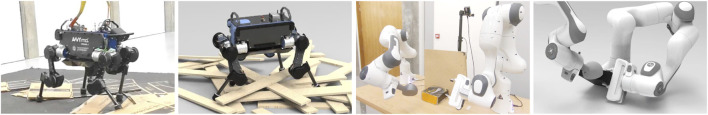
Robustness under uncertainties and generalisation for locomotion (left) and manipulation (right) both on real robots and in unseen test settings.

For validating how robust and versatile MES is, we deployed the multi-expert policies in test scenarios that have never been seen during training (see Figure 12, accompanying video). Specifically, we tested the policy under environmental and hardware uncertainties. The policies’ ability to robustly function in unseen environments and the successful transfer from simulation to the real world demonstrate that the learned MES policy is robust and can generalise across environments and domains with varying physics and dynamics properties.

#### 5.5.1 Environmental uncertainty

The MES policies were tested under varying dynamics parameters from 50% to 150% of the mass and inertia of the robot links. For locomotion, the robot traversed test terrains consisting of a cluster of planks, slippery objects (Figure 12 left), and withstood large pushes on the real robot (see accompanying video).

For dual-arm manipulation, the environment was modified by using different objects and support bases. We replaced the flat wall with the grasper’s base as support (Figure 12 right) and a round wall (see video). We replaced the nominal box with a torus (see video) as the object. Despite altering the environment in which the robot interacts with different objects and support walls, the policy can still adapt and complete the task. Furthermore, the successful dual-arm cooperation was shown in real experiments (Figure 12 right), and under disturbances applied on the object (see accompanying video).

#### 5.5.2 Hardware discrepancies

The multi-expert policy robustly completes the task under hardware uncertainties from actuators, sensors, and varying dynamic parameters, such as inertia and mass (see accompanying video). For both actuators and sensors, we tested three settings in simulation: adding Gaussian noise to the signal, setting the signal to zero, and randomizing the signal uniformly. Such setting corresponds to the actuators being corrupted by noise, jammed in a zero position, or receiving jerky commands. For the sensors, the measurements become noisy, zero, and erroneous respectively.

Despite the existence of hardware discrepancies, the multi-expert policy achieved stable trotting and completed lifting and grasping of the object. This shows the robustness of MELA as a feedback policy to realise different motor tasks, i.e., quadruped locomotion and bimanual manipulation.

## 6 Comparison of multi-expert learning architecture and mixture of experts

MoE approaches have been reported to scale poorly for control of a high degree-of-freedom systems (Zhang et al., 2018; Yang et al., 2020b). The limited expressiveness of the low-dimensional latent space, i.e., the action space, causes an imbalance in expert behaviour that favours some experts and downgrades others (Shazeer et al., 2017; Zhang et al., 2018).

### 6.1 Task performance

**FIGURE 13:**
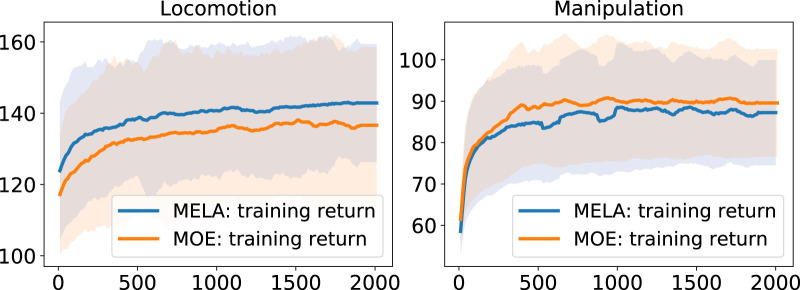
Comparison of learning curves of MoE and MELA. The learning curves are averaged over 5 separate training runs and the robot can stand with a reward higher than 100.

We compared MELA with MoE based on the task performance in quadruped locomotion and dual-arm cooperation. The learning curves can be seen in Figure 13.

To analyse the transferability and robustness of MELA and MoE across domains, we validated the robust quadruped policy in a different physics simulation, i.e., a simulation to simulation (sim2sim) transfer from pybullet to Gazebo. Gazebo simulator was chosen because the same software infrastructure was used for running the real robot Anymal. The practical and common discrepancies in real world experiments that can cause poor policy performance (Peng et al., 2018b; Tan et al., 2018; Hwangbo et al., 2019) were introduced in Gazebo, including the mismatch in the physics model, signal noises, feedback latency, friction and damping, and drift in sensory measurements. All quantities used by the MES policies were directly measured and filtered from the robot, or obtained through the state estimation algorithm that runs on the real robot.

**FIGURE 14:**
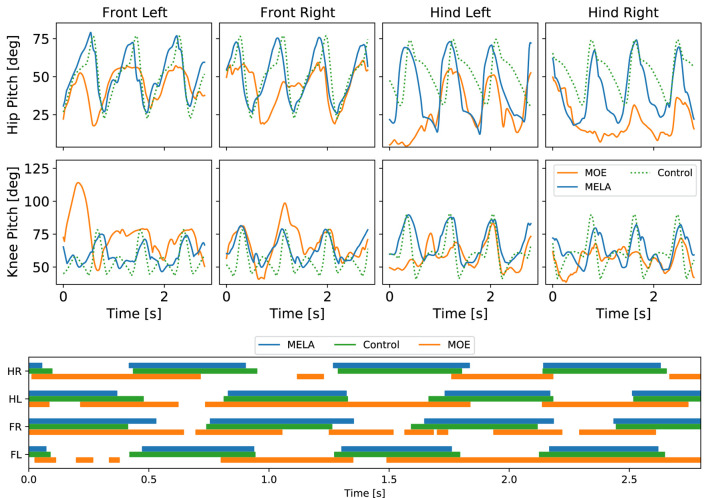
Locomotion comparison between control, MoE and MELA. Top: Joint position. Bottom: Gait pattern of Front Left (FL), Front Right (FR), Hind Left (HL), Hind Right (HR) feet.

While both MoE and MELA can learn robust locomotion in the PyBullet simulation, only MELA was able to transfer the learned policy and perform successful trotting in both a different simulator Gazebo and the real robot. In contrast, MoE was not able to reproduce successful trotting across a different physics simulation or real system and environment, which was shown by a downgraded behavior of a dragging leg that caused a complete fall (see Figure 14 and accompanying video).

In addition to the literature (Shazeer et al., 2017; Zhang et al., 2018; Peng et al., 2019), our comparison finds that the low-dimensional latent space of MoE lacks the complexity for encoding sufficient features robust against physical variations, which is needed for a domain transfer of tasks, such as gait recovery and trotting.

In Figure 14, the qualitative differences and similarities among baseline control, MoE and MELA are shown. The baseline control data was used as imitation reference for both MELA and MoE. The average difference of joint positions between baseline control and MELA was 5°, while MoE deviated by 13°. The periodicity and trotting pattern is noticeable in both MELA and baseline control, while MoE was could not reproduce the periodic gait pattern.

### 6.2 Diversity of skills

We analysed the diversity of skills of MELA and MoE by a t-distributed Stochastic Neighbor Embedding (t-SNE) analysis (Maaten and Hinton, 2008). T-SNE projects high-dimensionals NN activation on a 2D plane by clustering similar NN activations together but keeping dissimilar data points distant, which can be used to analyse robotic behaviours (Yuan et al., 2020).

**FIGURE 15:**
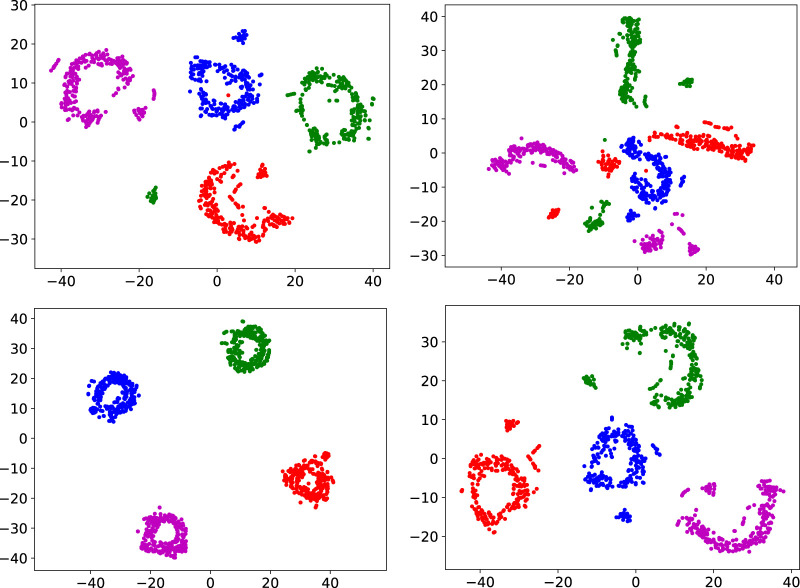
Comparison of the t-SNE analysis between MELA and MOE with and without the proposed diversity enforcement, using 4 experts for locomotion (blue and purple) and gait recovery (red and green). Top left: MELA without diversity enforcement, top right: MOE without diversity enforcement, bottom left: MELA with diversity enforcement, bottom right: MOE with diversity enforcement.

The experts’ neuron activations (all N experts in Figure 4) during time-step *k* were stacked as one high-dimensional data point $h_{k}^{i}\in R^{\left(256+256+12\right)}$ for all *i* experts. During one rollout of 250 time steps, 250 data points $h_{k}^{i}$ were collected to produce the t-SNE analysis shown in Figure 15.

Figure 15 shows a t-SNE analysis comparing MELA and MoE with and without the diversity term (19). From the distinct clusters and separation distances (Figure 15 bottom left and right), the diversity among experts using our technique can be seen. MELA shows clustering without the diversity term (Figure 15 top left) and has more distinct expert clusters using the diversity term. For MoE, the experts collapse to one indistinguishable cluster if no diversity term is used (Figure 15 top right) and form four clusters when diversity is enforced (Figure 15 bottom right).

## 7 Conclusion and future work

In this work, we proposed: 1) a Multi-Expert Synthesis (MES) framework that can generate motor skills by synthesising expert skills, which is applicable for both robot locomotion and manipulation; 2) an automatic algorithm of selecting relevant physical variables for the state observations of reinforcement learning; and 3) techniques to augment networks and enforce diversify of experts which address the expert imbalance problem in multi-expert approaches.

Both simulation and experiments showed that MES can learn robust quadrupedal locomotion by combining the skills of gait recovery and trotting. Further, MES demonstrated dual-arm manipulation and grasping, where one robot arm pregrasped an object and changed it to a feasible grasp pose, and the other robot arm grasped the object. The robustness of the learned MES policies were rigorously tested by a range of tests in both simulation and real world experiments, which have not been seen during training.

We evaluated two different MES approaches for locomotion and the results were analysed in terms of gait patterns and diversity of experts using a t-SNE analysis. The analysis suggested that our proposed algorithm for state selection was effective allowing locomotion that exhibits the typical gait patterns of quadrupeds, and that our proposed technique enforcing skill diversity between experts indeed removes expert imbalance.

In future work, we plan to expand the MES structure to incorporate visual perception information to allow robot motions that rely on visual inputs. Furthermore, we intend to learn multi-expert policies that combine experts of different morphologies to control various robots with a unified policy.

## Data Availability

The raw data supporting the conclusions of this article will be made available by the authors, without undue reservation.
