# Supplementary material for: Multi-expert synthesis for versatile locomotion and manipulation skills
Source: Front Robot AI. 2022 Sep 28;9:970890. doi: 10.3389/frobt.2022.970890 (PMC9554355; doi:10.3389/frobt.2022.970890)
Supplement: Supplementary file 1 [file DataSheet1.pdf]

Multi-Expert Synthesis for Versatile Locomotion and Manipulation Skills

Kai Yuan [1], and Zhibin Li [2,*]

[1]School of Informatics, The University of Edinburgh, 47 Potterrow, EH8 9BT Edinburgh, United Kingdom

[2]Department of Computer Science, University College London, Gower St, London WC1E 6EA, United Kingdom

Correspondence*:
Corresponding Author
alex.li@ucl.ac.uk

1 PARAMETERS

Table 1. Variations of dynamics randomisation for training.

	Default value	Min (training)	Max (training)	Min (testing)	Max (testing)
Contact friction	0.7	50%	150%	30%	170%
Joint torque	40Nm	80%	120%	80%	200%
Inertia	link dependent	80%	120%	50%	150%
Mass	link dependent	80%	120%	50%	150%

Table 2. Reward weights for quadruped experts.

	w_{task}:	x_{vel}	y_{vel}	z_{vel}	z_{pos}	g^L	reg	w_{imit}:	q	$\dot{q}$	cont.	eef pos
r_{gr}	1.0	1	1	1	5	10	1	0.0				
r_{stand}	1.0	2	2	2	4	4	1	0.0				
r_{loco}	0.3	6	1	0	1	3	1	0.7	0.5	0.2	0.05	0.25
